# Identification of factors predicting scar outcome after burn injury in children: a prospective case-control study

**DOI:** 10.1186/s41038-017-0084-x

**Published:** 2017-07-03

**Authors:** Hilary J. Wallace, Mark W. Fear, Margaret M. Crowe, Lisa J. Martin, Fiona M. Wood

**Affiliations:** 1 Burn Injury Research Unit, Faculty of Health and Medical Sciences, University of Western Australia, Perth, WA Australia; 2 Burns Service of Western Australia, Princess Margaret Hospital for Children and Fiona Stanley Hospital, Perth, WA Australia; 3 Burn Injury Research Unit, Faculty of Health and Medical Sciences, University of Western Australia, M318, 35 Stirling Highway, Crawley, 6009 WA Australia

**Keywords:** Burns, Wound healing, Hypertrophic scar, Risk factors, Children

## Abstract

**Background:**

There is a lack of rigorous research investigating the factors that influence scar outcome in children. Improved clinical decision-making to reduce the health burden due to post-burn scarring in children will be guided by evidence on risk factors and risk stratification. This study aimed to examine the association between selected patient, injury and clinical factors and the development of raised scar after burn injury. Novel patient factors were investigated including selected immunological co-morbidities (asthma, eczema and diabetes type 1 and type 2) and skin pigmentation (Fitzpatrick skin type).

**Methods:**

A prospective case-control study was conducted among 186 children who sustained a burn injury in Western Australia. Logistic regression was used to explore the relationship between explanatory variables and a defined outcome measure: scar height measured by a modified Vancouver Scar Scale (mVSS).

**Results:**

The overall correct prediction rate of the model was 80.6%; 80.9% for children with raised scars (>1 mm) and 80.4% for children without raised scars (≤1 mm). After adjustment for other variables, each 1% increase in % total body surface area (%TBSA) of burn increased the odds of raised scar by 15.8% (95% CI = 4.4–28.5%). Raised scar was also predicted by time to healing of longer than 14 days (OR = 11.621; 95% CI = 3.727–36.234) and multiple surgical procedures (OR = 11.521; 1.994–66.566).

**Conclusions:**

Greater burn surface area, time to healing of longer than 14 days, and multiple operations are independently associated with raised scar in children after burn injury. Scar prevention strategies should be targeted to children with these risk factors.

## Background

With the improved survival of children with major burns, prevention of hypertrophic scarring is an important focus of clinical burns care and research. Decisions about wound management are made to obtain good mortality and morbidity outcomes, including optimum scar outcome. Hypertrophic scars are raised scars confined within the boundaries of the wound and may cause considerable functional and cosmetic problems leading to limited range of motion and impaired psychosocial well-being [1]. Burn scars can prevent a child’s early return to school [2], and attitudes and beliefs towards disfigurement can lead to prejudice and discrimination at school and in the community [3]. ‘Scarring—a permanent reminder’, was a theme that emerged from a study exploring the psychological experiences of children following burn injury [4]. Improved clinical decision-making to reduce the health burden due to post-burn scarring in children will be guided by evidence on risk factors to identify children at high risk of poor scar outcome.

The reported prevalence of hypertrophic scarring after burn injury in children varies from 32 to 65% [5–7]. There is difficulty comparing results between existing studies due to different denominators and sample sizes, heterogeneous patient populations, a lack of consistent definitions of risk factors and a lack of consistent and valid scar outcome classification [8]. Despite the high prevalence of hypertrophic scarring as a complication of burn injury, few prospective studies have been conducted to systematically identify factors associated with their occurrence in children [9], and hypertrophic scars and keloids are not always well-differentiated [10].

This study aimed to examine the association between selected patient, injury and clinical factors and the development of raised scar after burn injury in children in a prospective study using a defined scar outcome measure. Novel factors were investigated including selected immunological co-morbidities (asthma, eczema and diabetes type 1 and type 2) and skin pigmentation (Fitzpatrick skin type).

A prospective case-control study was conducted among 186 child subjects who sustained a burn injury in Western Australia and were treated at Princess Margaret Hospital for Children. The primary outcome measure for the study was the scar height (SH) sub-score of the subjects’ worst scar according to a modified Vancouver Scar Scale (mVSS) [11]. We developed an epidemiological model for raised scarring after burn injury and report strength of association statistics (odds ratios and 95% confidence intervals) for factors associated with raised scarring which will assist individualised patient management.

## Methods

### Subjects

This case-control study was conducted at the Princess Margaret Hospital for Children (Western Australia), and subjects were recruited from December 2011 to July 2015. The research was conducted in accordance with Chapters 3.2 and 4.2 of the National Statement on Ethical Conduct in Human Research 2007 (National Health & Medical Research Council, Australia) and with approval of the Princess Margaret Hospital for Children Human Research Ethics Committee (Registration Number 1926/EP). The child’s participation required written consent of one parent (or where applicable the guardian or other primary care giver). For children mature enough to understand age-appropriate information (approximately 7 years of age and above), written assent was required.

Subjects were eligible for recruitment if they sustained an acute burn injury requiring hospital admission, outpatient treatment or hypertrophic scar treatment at Princess Margaret Hospital for Children and were 15 years of age or under at the time of their burn injury. All subjects were recruited in the outpatient clinic. Subjects were excluded from the study for the following reasons: parent or guardian unable to provide written informed consent, teenagers and primary school-age children unable to provide assent, a history of more than one hospital admission for acute burn injury, treatment with autologous cells harvested with ReCell^®^ device without a split-thickness skin graft, treatment with Integra^®^ Dermal Regeneration Template, acute burn injury treated outside Western Australia, previous history of keloid scarring, or burn scar diagnosed as a keloid scar.

### Patient treatment algorithm

**Fig. 1:**
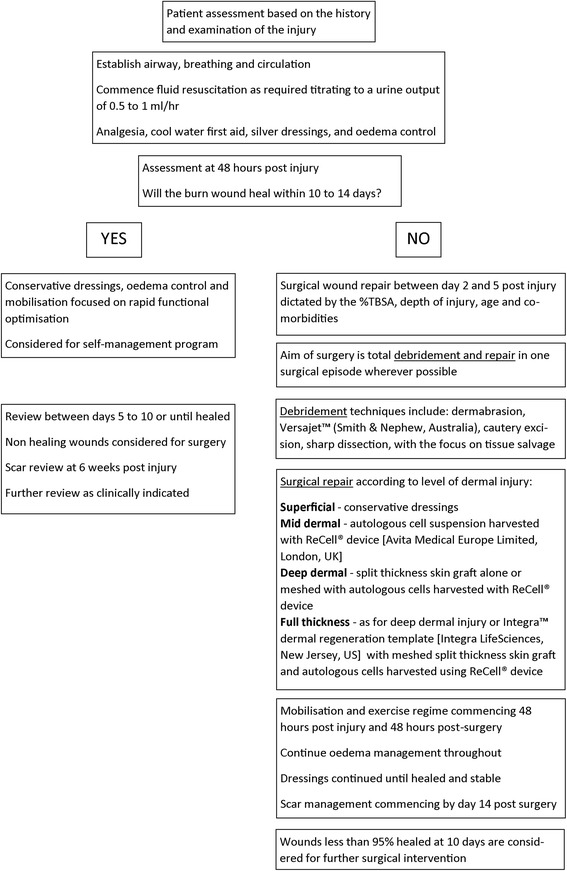
Patient treatment algorithm: optimal clinical treatment pathway for patients with burn injury in the care of the Burns Service of Western Australia

The clinical treatment pathway for children with burn injury in the care of the Burns Service of Western Australia is described in Fig. 1.

### Explanatory variables

At the time of recruitment, data for each subject on the following variables was extracted from medical records: age (at time of injury), sex, external cause of burn injury (scald, contact, flame, other), co-morbidities (asthma, eczema, diabetes type 1 or type 2), anatomical site of injury (head/neck, chest/abdomen, back/buttocks, arm, hand, leg, foot, genitalia), % total body surface area (%TBSA) of burn, length of hospital stay (days), surgery level (proxy variable for wound depth: conservative; split-thickness skin graft (SSG) ± autologous cells harvested with ReCell® device [Avita Medical Europe Limited, London, UK]), wound complications (yes/no: skin graft loss, over-granulation or wound infection), multiple surgical procedures (yes/no: more than one SSG procedure for an acute burn wound), healed within 14 days (yes/no: discontinuation of therapeutic dressings or statement that all wounds have healed within 14 days of burn injury [conservative] or first surgical procedure for acute burn wound [SSG ± autologous cells]). The Fitzpatrick skin type assessment was conducted separately by questionnaire using the Fitzpatrick Classification Scale [12] (Table 1).

**Table 1:** Fitzpatrick Skin Type Classification Scale categories [12]

Skin type	Skin colour	Characteristics
1	White; very fair; red or blonde hair; blue eyes;	Always burns, never tans
2	White; fair; red or blonde hair; blue, hazel or green	Usually burns, tans with difficulty
3	Cream white; fair with any eye or hair colour	Sometimes mild burn, gradually tans
4	Brown; typical Mediterranean Caucasian skin	Rarely burns, tans with ease
5	Dark brown; Middle Eastern skin types	Very rarely burns, tans very easily
6	Black	Never burns, tans very easily

### Primary outcome measure

**Fig. 2:**
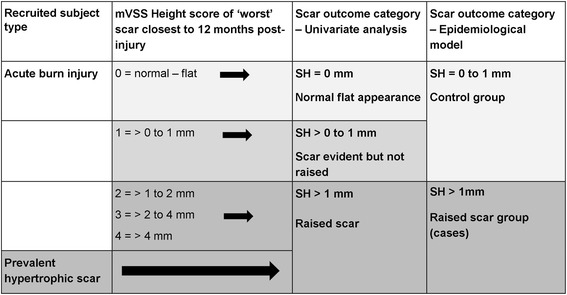
Flow-chart for primary scar outcome categories

Children with acute burn injury were followed up for 12 months post-injury with scar assessments scheduled at 3, 6 and 12 months using the mVSS [11, 13]. The primary outcome measure was the mVSS height sub-score (SH) of the subject’s ‘worst’ scar (scar area with the highest total mVSS score) closest to 12 months post-injury. The anatomical site of this scar was also recorded. Cut-off scores based on the SH measurement created three ordered categories for univariate analysis: normal flat appearance (SH = 0 mm); scar evident but not raised (SH > 0 to 1 mm); and raised scar (SH > 1 mm) (Fig. 2). Subjects with scar assessments discontinued prior to 12 months due to ‘excellent’ scar outcome in the clinical judgement of a consultant plastic surgeon were assigned to the normal flat category (SH = 0 mm). Subjects recruited with prevalent hypertrophic scarring (undergoing scar treatment with reconstructive surgery, intra-lesional steroids or laser therapy) were assigned to the raised scar category (SH > 1 mm). For the epidemiological model, the three categories were collapsed into two groups so that the model’s parameters were easier to interpret for clinical application: subjects with SH 0 to 1 mm comprising the control group; subjects with raised scar (SH > 1 mm) comprising the case group.

### Statistical analysis

Data analyses and modelling were performed using SPSS Statistics version 22.0 for Windows (IBM Corp, New York, USA). Explanatory and outcome variables were initially summarised by surgery level (conservative; SSG ± autologous cells). Continuous variables were expressed as the median and interquartile range (IQR), and frequencies were tabulated for categorical variables. Differences between the two levels of surgical intervention were analysed by cross-tabulation (Pearson’s chi-square–categorical variables) and the Mann-Whitney test (continuous variables). The sample size was not sufficient to undertake a sub-group analysis of the SSG group to examine the specific impact of autologous cells harvested with the ReCell^®^ device.

The explanatory variables were then examined in relation to the three primary scar outcome categories. Univariate analysis was conducted using cross-tabulation (Pearson’s chi square–categorical variables) or Kruskal-Wallis test (continuous variables). The epidemiological modelling used the collapsed dichotomous outcome measure (Fig. 2), and logistic regression was employed to estimate the probability of raised scar based on the values of the explanatory variables (Table 2). Approximately 65% of subjects were in the control group, and the case group data (subjects with raised scar) were weighted 2:1 to improve balance in the analysis. Initially all factors from the univariate analyses with a *p* value less than 0.15 were entered simultaneously into the logistic regression model. Backwards elimination of selected variables was performed for those that were the least significant (and with a *p* value greater than 0.05). All two- and three-way interactions of significant factors were examined to see if they significantly improved the model fit. The output provides measures of significance for each variable for prediction of scar outcome and estimates of increased risk for different levels of each factor in the form of odds ratios (with 95% confidence intervals) in comparison with a chosen reference level.

**Table 2:** Variables for inclusion in the logistic regression model

Variable description	Variable type	State description
Sex	Categorical	Female; male^a^
Age (years)	Continuous	
Fitzpatrick skin type	Categorical	1–3^a^; 4–6
History of asthma	Categorical	No^a^; yes
%TBSA	Continuous	
External cause of burn	Categorical	Scald^a^; contact; flame; other
Wound depth (proxy variable surgery level)	Categorical	Conservative^a^; Split-thickness skin graft ± ReCell®
Healed within 14 days	Categorical	No; yes^a^
Wound complications (over-granulation, graft loss or wound infection)	Categorical	No^a^; yes
Multiple surgical procedures for acute wound (>1)	Categorical	No^a^; yes
Length of stay (days)	Categorical	0^a^; >0–7; >7–14; >14–30; >30–60; >60
Worst scar location	Categorical	Face/head/neck; chest/abdomen/groin; back/buttocks; arm; hand; leg^a^; foot
Time from injury to scar assessment (months)	Continuous	

^a^Reference state

## Results

### Study population

A total of 229 subjects were recruited. After the exclusion criteria were applied and removing subjects with missing scar outcome data, a total of 186 subjects were available for analysis. Most subjects (76.4%) were recruited during treatment of an acute burn injury; 23.6% were recruited during treatment for hypertrophic scarring resulting from a burn injury.

Males comprised just over half the study subjects (58.1%), and the median age overall was 5.3 years (IQR 1.9–10.5). Scalds were the predominant cause of injury (47.3%). Approximately one third of the subjects were treated conservatively (36.0%). Of the subjects who had a SSG, 81 (68.1%) also had an application of autologous cells harvested with the ReCell^®^ device, and the median time from injury to the first surgical procedure was 6.0 days (IQR 3.0–10.0).

Table 3 summarises the distribution of the explanatory variables according to the level of surgical intervention. There was no significant age or gender difference between the levels of surgical intervention. The median %TBSA of the SSG group (4.0%) was double than that of the conservative group (2.0%) (*p* = 0.005). There was no significant difference in the distribution of individual Fitzpatrick skin types, or proportion of subjects with asthma or eczema between subjects treated conservatively or with a SSG. No subjects had a history of type 1 or type 2 diabetes. In the conservatively treated group, there were a higher proportion of scalds (62.1 vs. 39.0%) and there were a higher proportion of flame burns in the SSG group (22.9 vs. 4.5%) (*p* = 0.003). There were no significant differences in anatomical burn location between the conservative group and the SSG group. In subjects treated conservatively, the proportion with wounds that healed within 14 days was double than the proportion observed in the SSG group (52.6 vs. 24.3%) (*p* < 0.0001). Conversely, the proportion of subjects treated conservatively who had wound complications was approximately half the proportion observed in the SSG group (23.1 vs. 46.4%) (*p* = 0.002). The SSG group had a significantly higher proportion of subjects with raised scar (42.9 vs. 19.4%) (*p* < 0.0001).

**Table 3:** Characteristics of the study population according to the level of surgical intervention

		Conservative (*n* = 67)		Split-thickness skin graft (*n* = 119)		Total (*n* = 186)		*p* value	Test
		*n*	%^a^	*n*	%^a^				
Gender	Females	31	46.3	47	39.5	78	41.9	0.369	Pearson’s chi-square (*df* = 1)
	Males	36	53.7	72	60.5	108	58.1		
Age	Median (IQR)	5.70 (2.40–12.10)		5.10 (1.80–10.00)		5.30 (1.90–10.50)		0.403	Mann-Whitney
TBSA (%)^b^	Median (IQR)	2.00 (1.00–5.00)		4.00 (1.50–8.00)		3.00 (1.00–7.00)		0.005	Mann-Whitney
Fitzpatrick skin type^c^	1	0	0	3	2.6	3	1.7	0.285	Pearson’s chi-square (*df* = 5)
	2	16	24.6	17	14.7	33	18.2		
	3	29	44.6	59	50.9	88	48.6		
	4	9	13.8	19	16.4	28	15.5		
	5	4	6.2	11	9.5	15	8.3		
	6	7	10.8	7	6.0	14	7.7		
Asthma^d^	Yes	5	7.8	17	14.3	22	12.0	0.199	Pearson’s chi-square (*df* = 1)
Eczema^e^	Yes	8	12.5	15	12.6	23	12.6	0.984	Pearson’s chi-square (*df* = 1)
Diabetes^f^ (type 1 or type 2)	Yes	0	0.0	0	0.0	0	0.0	N/A	Pearson’s chi-square (*df* = 1)
External cause of burn^g^	Flame	3	4.5	27	22.9	30	16.3	0.003	Pearson’s chi-square (*df* = 6)
	Contact	18	27.3	39	33.1	57	31.0		
	Scald	41	62.1	46	39.0	87	47.3		
	Sunburn or radiation	1	1.5	0	0.0	1	0.5		
	Chemical	0	0.0	0	0.0	0	0.0		
	Friction	3	4.5	6	5.1	9	4.9		
	Electrical	0	0.0	0	0.0	0	0.0		
Burn location	Head/neck	15	22.4	17	14.3	32	17.2	0.160	Pearson’s chi-square (*df* = 1)
	Chest/abdomen/groin	26	38.8	36	30.3	62	33.3	0.235	Pearson’s chi-square (*df* = 1)
	Back/buttocks	10	14.9	15	12.6	25	13.4	0.656	Pearson’s chi-square (*df* = 1)
	Arm	20	29.9	41	34.5	61	32.8	0.521	Pearson’s chi-square (*df* = 1)
	Hand	18	26.9	27	22.7	45	24.2	0.523	Pearson’s chi-square (*df* = 1)
	Leg	20	29.9	51	42.9	71	38.2	0.080	Pearson’s chi-square (*df* = 1)
	Foot	8	11.9	18	15.1	26	14.0	0.548	Pearson’s chi-square (*df* = 1)
	Genitalia	2	3.0	5	4.2	7	3.8	0.676	Pearson’s chi-square (*df* = 1)
Multiple surgical procedures	>1 surgical procedure	0	0.0	25	21.0	25	13.4	<0.0001	Pearson’s chi-square (*df* = 1)
Healed in 14 days^h^	Yes	30	52.6	25	24.3	55	34.4	<0.0001	Pearson’s chi-square (*df* = 1)
Wound complications^i^	Yes	15	23.1	52	46.4	67	37.9	0.002	Pearson’s chi-square (*df* = 1)
Length of stay^j^ (days)	Median (IQR)	2.00 (0.00–6.25)		12.00 (4.00–21.00)		7.00 (1.00–16.00)		<0.0001	Mann-Whitney
Scar height (SH)	0 mm (normal–flat)	40	59.7	20	16.8	60	32.3	<0.0001	Pearson’s chi-square (*df* = 2)
	>0 to 1 mm (scar evident but not raised)	14	20.9	48	40.3	62	33.3		
	>1 mm OR scar recon/steroids/laser (raised scar)	13	19.4	51	42.9	64	34.4		

^a^Column percentage

^b^Missing data, *n* = 1

^c^Missing data, *n* = 5

^d^Missing data, *n* = 3

^e^Missing data, *n* = 3

^f^Missing data, *n* = 3

^g^Missing data, *n* = 2

^h^Missing data, *n* = 26

^i^Missing data, *n* = 9

^j^Missing data, *n* = 3

### Univariate analysis–scar height

The SH outcomes of the subjects were spread evenly between the three categories (0, SH = 0 mm; 1, SH > 0 to 1 mm; 2, SH > 1 mm) (Table 4), with approximately 60 subjects per category. The median time from injury to scar assessment increased progressively with scar outcome category: 6.6 months (SH = 0 mm); 13.1 months (SH > 0 to 1 mm); and 34.4 months (SH > 1 mm) (*p* < 0.001). There was no significant difference between the proportion of males and females in the three scar outcome categories. Age was significantly different (*p* = 0.015) between the scar outcome categories, with a younger median age in the SH > 1 mm (3.8 years) and SH > 0 to 1 mm (4.25 years) categories compared to 8.2 years in the normal-flat (SH = 0 mm) category.

**Table 4:** Univariate analysis of factors in relation to scar outcome

Explanatory variable		Scar height =0 mm (*n* = 60)		Scar height >0 to 1 mm (*n* = 62)		Scar height >1 mm OR scar reconstructive surgery/steroids/laser (*n* = 64)		Total (*n* = 186)		*p* value	Test
		*n*	%^a^	*n*	%^a^	*n*	%^a^	*n*	%^a^		
Sex	Female	28	46.7	24	38.7	26	40.6	78	41.9	0.650	Pearson’s chi-square (*df* = 2)
	Male	32	53.3	38	61.3	38	59.4	108	58.1		
Age (years)	Median (IQR)	8.20 (3.48–12.58)		4.25 (1.70–9.15)		3.80 (1.55–9.22)		5.30 (1.90–10.50)		0.015	Kruskal-Wallis
Time from injury to scar assessment (months)^b^	Median (IQR)	6.60 (2.70–21.68)		13.10 (8.52–52.38)		34.40 (10.32–68.00)		13.25 (6.08–47.35)		<0.0001	Kruskal-Wallis
Asthma^c^	Yes	8	14.0	3	4.8	11	17.2	22	12.0	0.088	Pearson’s chi-square (*df* = 2)
Eczema^d^	Yes	9	15.8	9	14.5	5	7.8	23	12.6	0.355	Pearson’s chi-square (*df* = 2)
Diabetes (type 1 or type 2)	Yes	0	0.0	0	0.0	0	0.0	0	0.0	N/A	Pearson’s chi-square (*df* = 2)
Fitzpatrick skin type^e^	1–3	17	29.8	18	29.5	22	34.9	57	31.5	0.768	Pearson’s chi-square (*df* = 2)
	4–6	40	70.2	43	70.5	41	65.1	124	68.5		
% TBSA^f^	Median (IQR)	3.00 (1.00–5.00)		3.00 (1.00–7.25)		4.00 (1.50–9.75)		3.00 (1.00-7.00)		0.018	Kruskal-Wallis
Level of surgical intervention (proxy for wound depth)	Conservative	40	66.7	14	22.6	13	20.3	67	36.0	<0.0001	Pearson’s chi-square (*df* = 2)
	Split-thickness skin graft	20	33.3	48	77.4	51	79.7	119	64.0		
Multiple surgical procedures (>1)	Yes	0	0.0	5	8.1	20	31.3	25	13.4	<0.0001	Pearson’s chi-square (*df* = 2)
Healed within 14 days^g^	Yes	33	63.5	19	30.6	3	5.8	55	34.4	<0.0001	Pearson chi-square (*df* = 2)
Wound complications^h^	Yes	10	17.2	28	45.9	29	50.0	67	37.9	<0.0001	Pearson’s chi-square (*df* = 2)
Length of stay (days)^i^	Median (IQR)	3.00 (0.00–8.00)		6.50 (1.75–15.25)		14.00 (5.00–25.50)		7.00 (1.00–16.00)		<0.0001	Kruskal-Wallis
Worst scar location^j^	Head/neck	3	5.1	2	3.2	3	5.3	8	4.5	0.850	Pearson’s chi-square (*df* = 14)
	Chest/ abdomen	14	23.7	12	19.4	7	12.3	33	18.5		
	Back/buttocks	4	6.8	3	4.8	2	3.5	9	5.1		
	Arm	7	11.9	13	21.0	9	15.8	29	16.3		
	Hand	11	18.6	8	12.9	12	21.1	31	17.4		
	Leg	15	25.4	17	27.4	16	28.1	48	27.0		
	Foot	5	8.5	6	9.7	8	14.0	19	10.7		
	Genitalia	0	0.0	1	1.6	0	0.0	1	0.6		

^a^Column percentage

^b^Missing data, *n* = 10

^c^Missing data, *n* = 3

^d^Missing data, *n* = 3

^e^Missing data, *n* = 5

^f^Missing data, *n* = 1

^g^Missing data, *n* = 26

^h^Missing data, *n* = 9

^i^Missing data, *n* = 3

^j^Missing data, *n* = 1

There was no significant association between scar outcome and Fitzpatrick skin type (groups 1–3 vs. 4–6) or history of asthma or eczema. Subjects in the raised scar category (SH > 1 mm) had a greater median %TBSA than subjects with SH = 0 to 1 mm (4.00 vs. 3.00) (*df* = 2, *p* = 0.018). Only one third of the subjects in the normal-flat category (SH = 0 mm) were treated with a SSG compared to nearly 80% in the SH > 0 mm categories (*df* = 2, *p* < 0.0001). Of the subjects who underwent multiple surgical procedures (*n* = 25), 80% had a raised scar (SH > 1 mm) compared to 27.3% in subjects who did not undergo multiple surgical procedures (*df* = 2, *p* < 0.0001). The proportion of subjects who healed their wounds within 14 days decreased with increasing scar height, with nearly two thirds of subjects with normal-flat scars (SH = 0 mm) healed within 14 days (63.5%) compared to only 5.8% in the raised scar category (SH > 1 mm) (*df* = 2, *p* < 0.0001). Conversely, while approximately half of the subjects with SH > 0 mm were reported to have a wound complication, the proportion of complications in the normal-flat category (SH = 0 mm) was only 17.2% (*df* = 2, *p* < 0.0001). There was no significant difference in the distribution of scar outcomes according to the anatomical location of the worst scar.

**Fig. 3:**
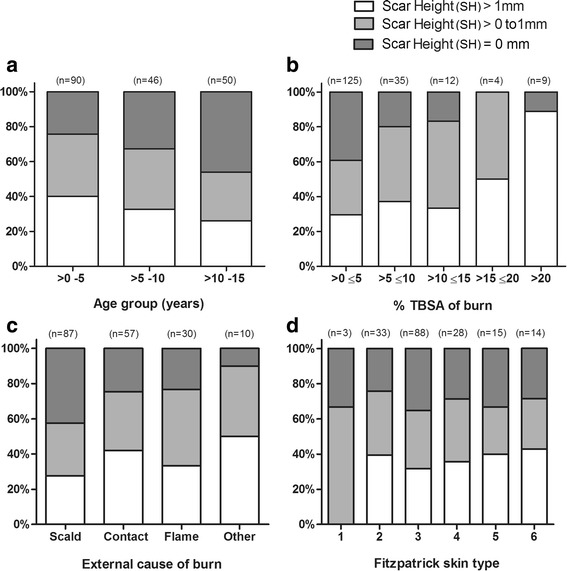
Distribution of primary outcome (scar height (SH)) according to patient and clinical characteristics. **a** Age group (NS; *p* = 0.130). **b** %TBSA of burn injury (*p* = 0.006). **c** External cause of burn (NS; *p* = 0.108). **d** Fitzpatrick skin type (NS; *p* = 0.931). *NS* not significant. Statistical test: Pearson’s chi-square

The distribution of scar outcomes for categorical variables with multiple levels is shown in Figure 3. The proportion of subjects in each scar outcome was not significantly different between age groups 0–5 years, >5–10 years and >10–15 years. The proportion with raised scar was significantly different between %TBSA categories, increasing from 15% TBSA (*df* = 8, *p* = 0.006). In univariate analysis, the difference in scar outcome between external causes of burn and Fitzpatrick skin types was not significant.

### Logistic regression model–scar height

Results of the logistic regression indicated that the nine-predictor model provided a statistically significant improvement over the constant-only-model, *χ* ^2^(18, *N* = 154) = 107.451, *p* < 0.0001. The Wald tests showed that three variables significantly improved the prediction of raised scar outcome after adjustment for other variables (Table 5): %TBSA, healed within 14 days and multiple surgical procedures. In the initial fitting of the model, wound complications, history of asthma, Fitzpatrick skin type, length of stay and time from injury to scar assessment were found to be not significant and were removed from the final model. The final model included adjustment for age (years), sex, level of surgical intervention, ‘worst’ scar location, external cause of burn and the interaction between external cause of burn and level of surgical intervention. These variables were retained to adjust for their potential effects on scar outcome and on the basis of the model diagnostics for classification accuracy and goodness of fit. The Nagelkerke pseudo *R* ^2^ indicated that the model accounted for 55.3% of the total variance and is likely to be a reasonable predictor of the outcome for any particular individual child [14]. A classification table was used to evaluate the percentage of correct predictions for each possible outcome according to the model. The overall correct prediction rate was 80.6%; 80.9% for those with raised scar and 80.4% for those without raised scar. The model’s goodness of fit assessed by the Hosmer-Lemeshow test (*p* = 0.225) indicated that the model was correctly specified (close match between the predicted frequencies and observed frequencies) [15].

**Table 5:** Logistic regression model for prediction of raised scar outcome

	Wald	*df*	*p* value	Odds ratio	95% CI for odds ratio	
					Lower	Upper
Age (years)	0.580	1	0.446	1.044	0.935	1.165
%TBSA	7.669	1	0.006	1.158	1.044	1.285
Sex						
Female sex^a^	0.008	1	0.928	1.041	0.436	2.484
Level of surgical intervention (proxy for wound depth)						
SSG ± ReCell^®b^	1.558	1	0.212	0.446	0.126	1.584
Healed within 14 days						
Not healed within 14 days^c^	17.873	1	0.000	11.621	3.727	36.234
Multiple surgical procedures						
Multiple surgical procedures^d^	7.459	1	0.006	11.521	1.994	66.566
External cause of burn	2.759	3	0.430			
External cause of burn × Level of surgical intervention (interaction)	7.169	3	0.067			
Worst scar location	8.476	6	0.205			
Constant	12.758	1	0.000	0.016		

Odds ratios and confidence intervals for the association between patient, injury and clinical factors and risk of developing raised scar after burn injury (total *n* = 186; missing cases in this analysis = 32)

^a^Reference state = male

^b^Reference state = conservative treatment

^c^Reference state = healed within 14 days

^d^Reference state = 0 or 1 surgical procedures

According to the logistic regression model, the odds of a child having a raised scar increase by 15.8% for each 1% increase in %TBSA (*p* = 0.006). With respect to clinical factors, children who take longer than 14 days to heal a wound have 11.6 times the odds of developing raised scar compared to those who heal within 14 days (*p* < 0.0001), and those who undergo multiple surgical procedures have 11.5 times the odds of developing raised scar compared to those without multiple surgical procedures (*p* = 0.006). As shown in Table 5, there are wide confidence intervals around the odds ratio estimates for healing within 14 days and multiple surgical procedures.

## Discussion

There is a lack of rigorous research investigating the factors that influence scar outcome in children. Using a prospective study design and logistic regression, we have identified three factors that are associated with SH > 1 mm in children after burn injury: greater %TBSA (burn size), healing time greater than 14 days and multiple surgical procedures. Two of the main factors previously linked to scar outcome in children relate to the severity of the burn injury—burn depth and burn size. Burn depth has been shown to influence overall scar quality (for example, POSAS observer score) [9, 16] and scar thickness [17]. In this study, the level of surgical intervention was used as a proxy marker of burn depth; the level of surgical intervention was not a significant factor in the model, suggesting that other associated variables, for example, healing within 14 days, multiple surgical procedures, %TBSA and external cause of burn (see Table 3) accounted for some of the difference in scar outcome. This is consistent with the results of Gangemi and colleagues [18], but a recent study conducted in adults in a larger sample found level of surgical intervention was significant after adjustment for other variables [19]. The use of the level of surgical intervention as a proxy marker of burn depth is also confounded by the direct impact of the intervention on scar outcome, with the SSG treatment protocol used in this hospital setting (68.1% in conjunction with autologous cells harvested with the ReCell^®^ device) possibly limiting the development of raised scar.

Burn size (%TBSA) was confirmed to be an important predictor of raised scar outcome in children in this study after adjustment for all other variables, with 1.158 (15.8%) increased odds of raised scar for each 1% increase in %TBSA. This translates to approximately twofold increased odds of raised scar for every 5% increase in %TBSA. The association between %TBSA and raised scar outcome is consistent with previous studies in children [9] and adults [19–21].

This study also confirmed earlier studies (univariate analyses) showing that delayed epithelialization beyond 10 to 14 days increases the incidence of hypertrophic scarring [5, 22, 23]. Chipp and colleagues showed that the risk of hypertrophic scarring was multiplied by 1.138 for every additional day beyond 8 days taken for the burn wound to heal [23]. Similarly, our study found that the increased odds of raised scar outcome in children with wounds that take longer than 14 days to heal is over 10-fold. This result, which adjusts for the effects of other variables, confirms that achievement of rapid wound closure is vital not only to minimise wound infection and life-threatening systemic sepsis but also to avoid excessive scar formation [24].

Multiple surgical procedures also predict raised scar outcome in children after adjusting for other variables, consistent with some other recent studies reporting that the number of operations is independently associated with hypertrophic scar severity in adults [21] and higher POSAS observer scores in adults and children [16] in regression analysis. In other studies conducted with adults [18, 19], multiple surgical procedures were significant in univariate analysis but not after adjustment for other variables.

This study agrees with the findings of another prospective study conducted in children (*n* = 284) which found no evidence that age, sex or the external cause of burn influenced scar quality (POSAS observer score) [9]. A recent study to identify risk factors for raised scar in adults [19] had a larger sample size (*n* = 636) and found that age and sex, but not the external cause of burn, were associated with raised scar outcome after adjustment for other factors. The lack of association between age and sex and scar outcome in the studies conducted in children to date may be a consequence of small sample sizes.

While Smith and colleagues [25] found that immunologic hypersensitivity or allergy was associated with the formation of hypertrophic scars, no association with asthma or eczema was demonstrated in this study even though approximately 12% of children in the study had a history of these conditions. Similarly, no association with raised scar was demonstrated for asthma or eczema in a study in adults [19]. Darker skin (Fitzpatrick skin types 4–6) was not significantly associated with raised scar, consistent with another paediatric study [23], in contrast to associations with darker skin types observed in adults [16, 19, 21]. Further studies are required to explore the relationship between skin pigmentation and raised scar outcome in children.

Complicating factors such as bacterial colonisation and infection of the wound are also suggested to induce hypertrophic scarring [26]. Our data showed an association between wound complications (graft loss, wound infection or excessive granulation) and raised scar outcome in univariate analysis, but not in the logistic regression model. This may be due to confounding with other variables, in particular, healing within 14 days and multiple surgical procedures.

A key strength of the study is the use of a defined outcome measure, SH, a quantitative, reliable and specific measure of hypertrophic scarring [13, 27] not confounded by scar vascularity and pigmentation. The prospective study design and the good performance measures of the model are also strengths of the study and support the validity of the results. The overall correct prediction rate of the model was 80.6%; 80.9% for children with raised scars (>1 mm) and 80.4% for children without raised scars (≤1 mm). The advantage of logistic regression is to avoid confounding effects by analysing the association of all variables together [28]. Most of the characteristics of our study population (age, sex distribution, external cause of burn injury, %TBSA and anatomical site burned) show a pattern of hospital admissions that is similar to many paediatric burns units, and surgical excision and skin grafting was generally performed early (median 6 days).

The sample size of this study (*n* = 186), although large in the context of many previously published studies on risk factors for hypertrophic scarring in children, is a limitation of this study. With the exception of %TBSA (continuous variable), only those factors with large odds ratios were detected as statistically significant and the confidence intervals were wide. An assessment of a factor as not significant should therefore not be considered definitive. A larger sample size is required to detect more subtle, but potentially important, factors that may influence scar height, to test interactions and to perform sub-cohort analyses (for example, the impact of autologous cells harvested with ReCell^®^). The study was not entirely ‘prospective’, with 23.6% of the subjects recruited with prevalent cases of hypertrophic scarring. The time from injury to scar assessment was not well controlled, which could lead to an over estimation of raised scar outcome in subjects with earlier outcome assessments [9]. However, in this study, there was no bias towards the raised scar group (SH > 1 mm) being assessed earlier than the control group (SH ≤ 1 mm), with median time from injury to scar assessment of 34.4 months (IQR 10.32–68.00) and 11.2 months (IQR 5.22–37.62) respectively. A limited number of variables was examined and there may be others that have an important bearing of the development of raised scar. For example, wounds subjected to tension (due to motion or body location) are consistently associated with risk of scar hypertrophy [29]. Some variables were collected at the subject level rather than scar level (e.g. multiple surgical procedures, wound complications, level of surgical intervention) which may have led to misclassification of the exposure for the ‘worst’ scar. Other variables were measured as categorical variables rather than continuous variables (for example healing within 14 days vs. healing time over 14 days) or collapsed into composite variables (for example ‘wound complications’ and ‘SSG ± autologous cells harvested with ReCell^®^ device’), which limited the sensitivity of the analyses. A proxy measure of burn depth was used in the study (level of surgical intervention), but a more direct assessment of burn depth would be the use of laser Doppler imaging [16, 30]. Another limitation of the study was the exclusion of mid-dermal burn injuries treated with autologous cells harvested with the ReCell® device (without SSG) and full-thickness burn injuries treated with Integra® Dermal Regeneration Template (with SSG) due to small numbers of subjects in these categories. The outcome measure used for the study, the height sub-score of a mVSS, is observer-dependent, and use of an objective device for measurement (for example high-frequency ultrasound) would further improve reliability and accuracy of the measurements [31].

## Conclusions

Using a logistic regression approach, this study provides further evidence on risk factors for raised scarring in children who have sustained a burn injury and will help guide decision-making. After adjustment for other variables, each 1% increase in burn %TBSA increased the odds of raised scar by 15.8%. Raised scar was also predicted by a healing time of greater than 14 days and multiple surgical procedures. Scar prevention strategies should be targeted to children with these risk factors. While the study was performed in a high-income country in a tertiary hospital setting, the consistency of results between existing studies suggests that these core risk factors may apply more generally. Due to the study limitations, the list of factors found to be non-significant should not be considered definitive. Similar to the conclusion of a recent systematic review on scar contractures [32], there is a need for more large-scale well-designed prospective studies with defined and harmonised outcome measures to explore further the risk factors for raised scar after burn injury in children.

## Abbreviations

CI: Confidence interval
OR: Odds ratio
POSAS: Patient and Observer Scar Assessment Scale
SH: Scar height
SSG: Split-thickness skin graft
%TBSA: % total body surface area
